# Survival in haemodialysis has improved considerably in Okinawa, Japan (1971–2020)

**DOI:** 10.1093/ckj/sfag284

**Published:** 2026-08-22

**Authors:** Takuhiro Moromizato, Ryoto Sakaniwa, Chiho Iseki, Akira Higa, Kentaro Kohagura, Kunitoshi Iseki

**Affiliations:** Okinawa Prefectural Nanbu Medical Centre and Children’s Medical Centre, Okinawa, Japan; Okinawa-Asia Clinical Investigation Synergy, Okinawa, Japan; Okinawa Prefectural Nanbu Medical Centre and Children’s Medical Centre, Okinawa, Japan; Public Health Department, Osaka University, Osaka, Japan; Okinawa-Asia Clinical Investigation Synergy, Okinawa, Japan; Nakamura Clinic, Clinical Research Support Centre, Urasoe, Japan; Okinawa-Asia Clinical Investigation Synergy, Okinawa, Japan; Okinawa Dialysis Physician Association, Naha, Japan; Shuri Jokamachi Clinic, Naha, Japan; Okinawa Dialysis and Transplant Association, Ginowan, Japan; Okinawa Dialysis and Transplant Association, Ginowan, Japan; Dialysis Unit, Ryukyu University Hospital, Ginowan, Japan; Nakamura Clinic, Clinical Research Support Centre, Urasoe, Japan; Okinawa Dialysis and Transplant Association, Ginowan, Japan; Okinawa-Asia Clinical Investigation Synergy, Okinawa, Japan

**Keywords:** dialysis vintage, end-stage kidney disease, haemodialysis, quality of life, survival trends

## Abstract

**Background:**

Dialysis care has advanced markedly over the past five decades; however, the extent of improvement and whether survival trends differ based on dialysis vintage remain unclear. We examined long-term mortality trends among patients on haemodialysis in Okinawa, Japan.

**Methods:**

We analysed 50-year registry data (1971–2020) from all dialysis facilities in Okinawa. Among the 15 698 adults who had undergone maintenance dialysis, outcomes were ascertained in 96.2%. Hazard ratios (HRs) for all-cause mortality were estimated using a Cox model. Standardized mortality ratios (SMRs) were calculated using age- and sex-specific mortality rates of the general Okinawan population. Patients were stratified by dialysis vintage at risk (<36 vs. ≥36 months), and Joinpoint analysis was used to identify temporal changes in SMR trends.

**Results:**

Overall survival improved steadily throughout the decades. Adjusted HRs for mortality declined to 0.43 (95% confidence interval, 0.38–0.49) in 2011–2020 from 1971 to 1980. However, the magnitude differed markedly with dialysis vintage. Among patients with a short vintage (<36 months), mortality declined to HR 0.21 in 2011–2020, whereas among those with a longer vintage (≥36 months; HR 0.67), improvement was attenuated. SMRs declined substantially until 2004, plateaued, then remained elevated in 2020.

**Conclusions:**

In this closed-cohort population, mortality among patients on dialysis declined markedly over five decades; survival gains were concentrated among patients with a short dialysis vintage, whereas improvements among long-vintage patients slowed in the 2000s. The persistent elevation of SMRs despite earlier improvements highlights the need for strategies targeting patients with prolonged dialysis exposure.

## Graphical Abstract

**Graphical Abstract:**
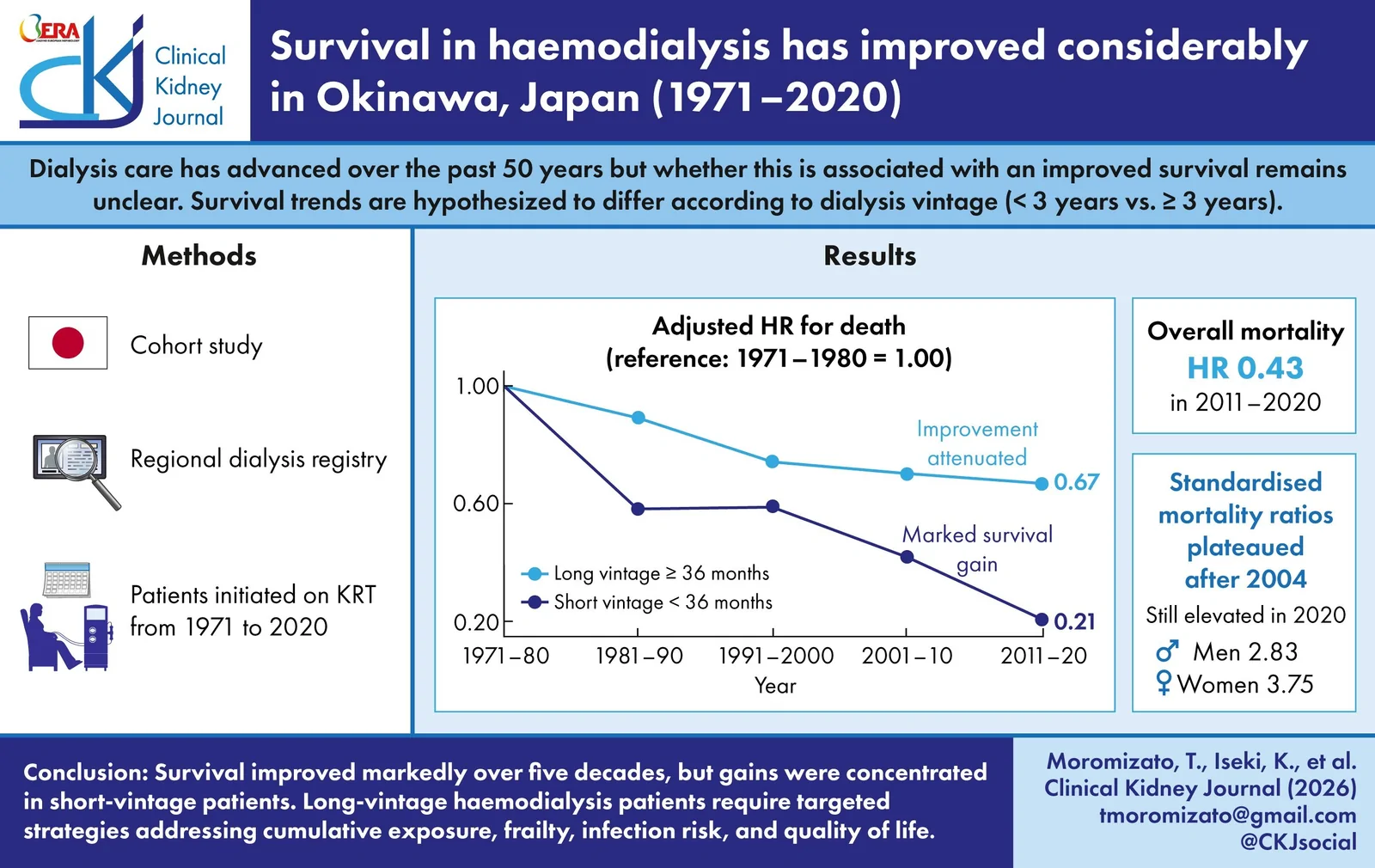

KEY LEARNING POINTS **What was known:**

- Despite major advances in dialysis technology, patients undergoing chronic dialysis continue to experience substantially higher mortality rates than the general population.
- Whether survival trends differ according to dialysis vintage remains unclear.

**This study adds:**

- Long-term mortality trends among patients on haemodialysis were examined in a geographically closed population over five decades.
- Mortality among patients on haemodialysis declined markedly over time.
- Survival gains were concentrated among patients with shorter dialysis vintage, whereas improvements among those with longer dialysis vintage slowed.

**Potential impact:**

- Incorporating dialysis vintage–stratified analyses into routine outcome surveillance, inter-regional benchmarking, and clinical trials may help identify meaningful disparities that could otherwise be masked.
- For patients receiving long-term haemodialysis, further improvements in survival may need to be considered alongside gains in quality of life.

## INTRODUCTION

More than half a century has passed since maintenance dialysis has become an established life-saving therapy for end-stage kidney disease in Japan [1–3]. Despite major advances in dialysis technology, infection control, anaemia management, and cardiovascular risk modification, patients undergoing chronic dialysis continue to experience substantially higher mortality rates than the general population [4]. Standardized mortality ratios (SMRs) remain elevated across countries and healthcare systems, highlighting the continued need to improve survival among dialysis populations [5, 6]. Understanding temporal changes in mortality is therefore essential for benchmarking care quality and identifying patient groups that have benefited least from therapeutic and healthcare system advances.

However, the impact of historical advances in dialysis care is unlikely to have been uniform across all patient subgroups [7]. Dialysis ‘vintage’, the duration a patient has already spent on dialysis, is a particularly important modifier of mortality risk [8, 9]. Early after dialysis initiation, survival is strongly influenced by acute illness burden and access to adequate treatment, whereas later survival increasingly reflects the complications of long-term dialysis exposure, multimorbidity, frailty, and treatment fatigue [10]. However, few studies have examined whether long-term survival trends differ between patients with short- vs. long-vintage dialysis and whether mortality improvements have been shared equally across these groups.

Okinawa, Japan, provides a unique opportunity to address this question. As a geographically isolated island with relatively limited migration, Okinawa functions as a population-based closed cohort, enabling near-complete longitudinal follow-up of patients on dialysis [1, 2]. Over the past five decades, all dialysis facilities in Okinawa have collaborated to maintain a comprehensive registry that captures outcomes across the entire treated population [10]. This rare setting allows evaluation of survival trends not only overall, but also within clinically meaningful subgroups, such as dialysis vintage.

In this study, using a 50-year population-based registry that included all dialysis patients in Okinawa, we examined long-term changes in survival and SMRs relative to the general population. We further assessed whether mortality trends differed between patients with short dialysis vintage (<36 months) and long dialysis vintage (≥36 months). We hypothesized that survival improvements would be greater among short-vintage patients, whereas gains among long-vintage patients may have slowed in recent decades. Clarifying these patterns may help identify priority populations and guide strategies to further improve survival in dialysis care.

## MATERIALS AND METHODS

### Study design and setting

We conducted a population-based cohort study using data from a comprehensive regional dialysis registry in Okinawa, Japan. Okinawa is an island prefecture with relatively limited migration, enabling long-term follow-up of a quasi-closed population. The study period spanned from 1 January 1971 to 1 January 2021. The STROBE reporting guidelines for observational cohort studies have been followed.

### Data source and registry development

Data were derived from the Okinawa Dialysis Study over 50 years (OKIDS50) registry, which includes all patients receiving renal replacement therapy (RRT) in Okinawa. The registry was developed in collaboration with all dialysis facilities across the prefecture [1, 2].

All data were anonymized prior to the analysis. The study protocol was approved by the Muribushi Clinical Training Centre Institutional Review Board (approval no. 2019-5) and conducted under an opt-out consent policy.

### Participants

All Okinawa residents who had undergone maintenance RRT (haemodialysis, peritoneal dialysis, or kidney transplantation) between 1971 and 2020 were included.

We excluded:

- patients treated exclusively for acute kidney injury;
- patients aged <18 years at RRT initiation;
- individuals who died or discontinued dialysis within 1 month of initiation; and
- those with incomplete baseline information.

Patients who relocated outside Okinawa were censored at their last confirmed treatment date. Kidney transplantation was treated as a censoring event. Recipients of pre-emptive transplantation were classified according to the transplant date and analysed separately.

### Variables

Baseline characteristics included age at dialysis initiation, sex, primary renal disease, and calendar year of initiation (grouped by decade).

Because dialysis duration is a strong determinant of prognosis, analyses were prespecified to stratify mortality risk based on dialysis vintage at the time of follow-up.

- short vintage: <36 months;
- long vintage: ≥36 months.

This categorization reflects clinically meaningful phases of dialysis exposure [10].

### Outcome

The primary outcome measure was all-cause mortality. Deaths were identified through medical record review, dialysis unit registries, and Japanese Society for Dialysis Therapy (JSDT) annual reports. Causes of death were coded into predefined clinical categories in the reports.

To compare the mortality with that of the general population, we estimated SMRs using age- and sex-specific mortality rates from the general Okinawan population for each calendar year.

### Statistical analysis

Follow-up continued until death, kidney transplantation, migration from Okinawa, loss to follow-up, or 1 January 2021 whichever occurred first.

Hazard ratios (HRs) and 95% confidence intervals (CIs) for mortality were estimated using Cox proportional hazards models adjusted for

- age at dialysis initiation;
- sex;
- diabetes mellitus;
- calendar period of dialysis initiation.

Temporal changes in the SMRs were evaluated using Joinpoint regression (National Cancer Institute Joinpoint Regression Program, version 5.3.0.0; Calverton, MD, USA) to identify statistically significant changes in mortality trends over time [11].

Pre-specified subgroup analyses were performed based on the dialysis vintage (<36 vs. ≥36 months) and sex. Kaplan–Meier curves were constructed for the decade of dialysis initiation.

Expected deaths were calculated using population census data for Okinawa (1975–2020) and mortality statistics from the Government of the Ryukyu Islands for 1970. Statistical analyses were conducted using STATA/SE (version 16.1; College station, TX, USA), with two-sided *P *< 0.05 considered statistically significant.

### Ethical considerations

This registry was established to improve dialysis care and patient outcomes in Okinawa, Japan. The requirement for individual informed consent was waived because the analyses used anonymized data with opt-out availability, which is consistent with the local ethical guidelines.

## RESULTS

### Study population and follow-up

**Figure 1:**
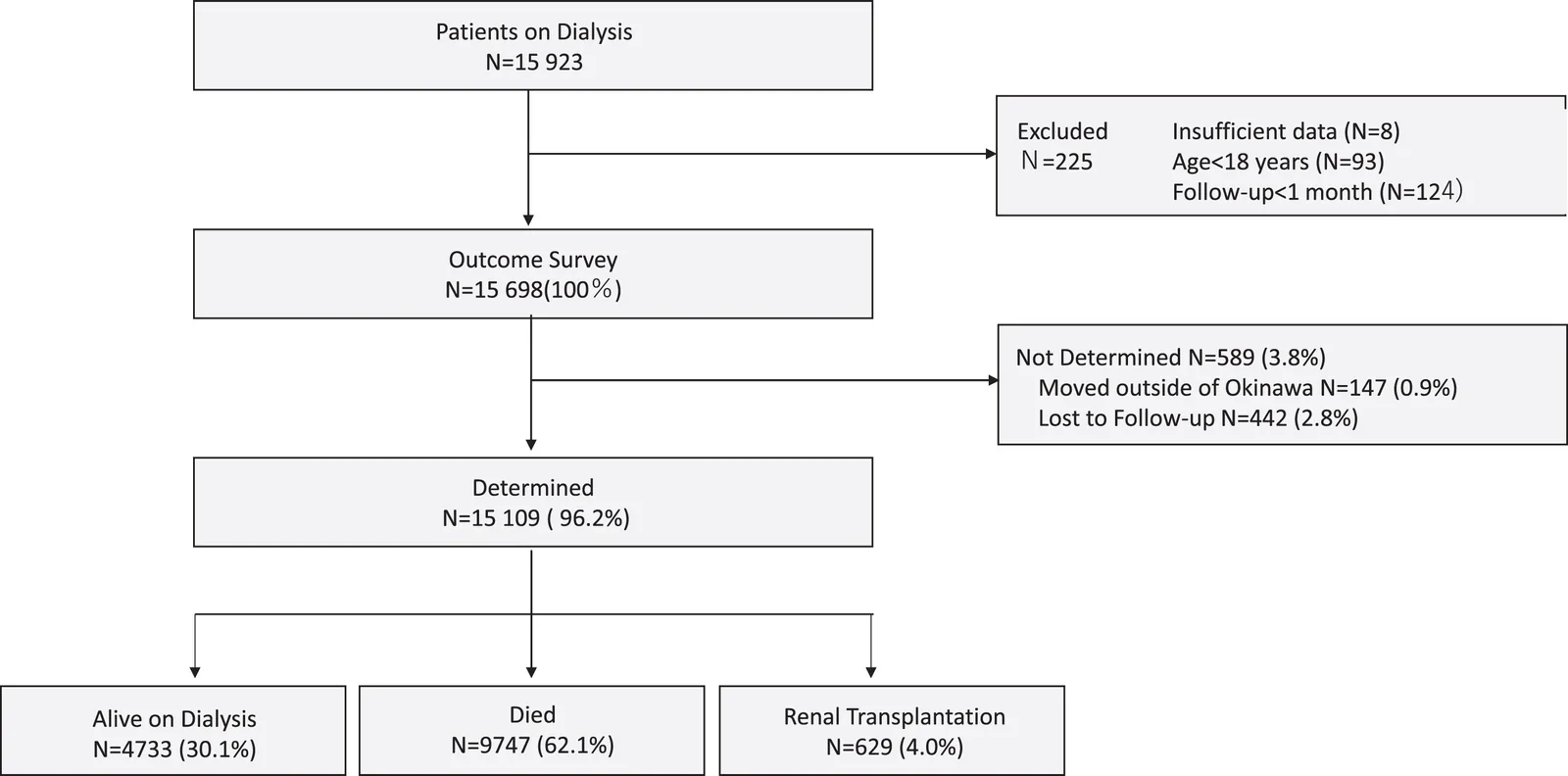
Flowchart of patient selection.

A total of 15 698 patients who initiated RRT in Okinawa between 1971 and 2020 were included in this study. The patient selection flowchart is shown in Fig. 1. Outcomes were successfully ascertained for 96.2% of the cohort. By the end of follow-up, 9747 patients (62.1%) had died, 4733 (30.1%) were alive and receiving dialysis, and 629 (4.0%) had undergone kidney transplantation. The demographic and clinical characteristics of the dialysis patients are presented in Table 1. The median survival duration was 4.8 years (interquartile range, 1.8–9.8 years).

**Table 1:** Demographics of patients on dialysis in Okinawa from 1971 to 2020.

	Total	Women	Men
Number (%) of patient	15 698 (100%)	6234 (40%)	9464 (60%)
Age at the start of dialysis, years			
Mean (SD)	62.2 (15.8)	64.1 (16.3)	60.9 (15.4)
Minimum–Maximum	18–101	18–99	18–101
Primary renal disease, number (%)			
Chronic glomerulonephritis	5488 (35.0%)	2258 (36.2%)	3230 (34.1%)
Diabetes mellitus	6335 (40.4%)	2332 (37.4%)	4003 (42.3%)
Nephrosclerosis	1579 (10.1%)	580 (9.3%)	999 (10.6%)
Polycystic kidney disease	303 (1.9%)	146 (2.3%)	157 (1.7%)
Systemic lupus erythematosus	199 (1.3%)	156 (2.5%)	43 (0.5%)
Others	1794 (11.4%)	762 (12.2%)	1032 (10.9%)
Outcomes, number (%)			
Alive on dialysis, 1 January 2021	4733 (30.2%)	1763 (28.3%)	2970 (31.4%)
Died	9747 (62.1%)	4020 (64.5%)	5727 (60.5%)
Renal transplantation	629 (4.0%)	236 (3.8%)	393 (4.2%)
Not determined	589 (3.8%)	215 (3.4%)	374 (4.0%)
Moved outside of Okinawa	147 (0.9%)	59 (0.9%)	88 (0.9%)
Lost to follow-up	442 (2.8%)	156 (2.5%)	286 (3.0%)
Cause of death, number (%)			
Infection	2642 (27.1%)	1090 (27.1%)	1552 (27.1%)
Malignancies	1268 (13.0%)	526 (13.1%)	742 (13.0%)
Heart failure, cardiac	1901 (19.5%)	726 (18.1%)	1175 (20.5%)
Sudden death	298 (3.1%)	93 (2.3%)	205 (3.6%)
Cerebrovascular disease	934 (9.6%)	370 (9.2%)	564 (9.8%)
Others	2704 (27.7%)	1215 (30.2%)	1489 (26.0%)
Total	9747 (100%)	4020 (100%)	5727 (100%)

### Overall changes in mortality over time

All-cause mortality declined progressively across the calendar periods. Compared with patients who initiated dialysis in 1971–1980, the adjusted hazard ratio (aHR) for death decreased to 0.79 in 1981–1990, 0.63 in 1991–2000, 0.53 in 2001–2010, and 0.43 in 2011–2020 (Table 2).

**Table 2:** Risk factors of death among patients on dialysis in Okinawa from 1971 to 2020.

	Total	<36 months	≥36 months
	HR (95% CI), *P*-value	HR (95% CI), *P*-value	HR (95% CI), *P*-value
Sex			
Men	1.00	1.00	1.00
Women	0.88 (0.84–0.92), *P *< 0.001	0.99 (0.93–1.06), NS	0.85 (0.81–0.89), *P *< 0.001
Age at the start of dialysis			
18–40 years old	1.00	1.00	1.00
41–60	2.69 (2.46–2.94), *P *< 0.001	1.47 (1.21–1.79), *P *< 0.001	2.73 (2.47–3.02), *P *< 0.001
61–80	7.39 (6.72–8.12), *P *< 0.001	2.05 (1.70–2.47), *P *< 0.001	7.48 (6.71–8.34), *P *< 0.001
81 and over	18.29 (16.39–20.41), *P *< 0.001	2.78 (2.28–3.38), *P *< 0.001	19.84 (17.21–22.89), *P *< 0.001
Year in which dialysis was started			
1971–1980	1.00	1.00	1.00
1981–1990	0.79 (0.71–0.89), *P *< 0.001	0.58 (0.46–0.71), *P *< 0.001	0.89 (0.78–1.03), NS
1990–2000	0.63 (0.56–0.71), *P *< 0.001	0.59 (0.48–0.72), *P *< 0.001	0.74 (0.64–0.86), *P *< 0.001
2001–2010	0.53 (0.47–0.59), *P *< 0.001	0.42 (0.35–0.52), *P *< 0.001	0.70 (0.60–0.81), *P *< 0.001
2011–2020	0.43 (0.38–0.49), *P *< 0.001	0.21 (0.17–0.25), *P *< 0.001	0.67 (0.58–0.79), *P *< 0.001
Primary renal disease			
Chronic GN	1.00	1.00	1.00
Diabetes mellitus	1.65 (1.57–1.74), *P *< 0.001	0.97 (0.89–1.05), NS	1.83 (1.72–1.94), *P *< 0.001
Nephrosclerosis	1.31 (1.22–1.41), *P *< 0.001	0.85 (0.75–0.96), *P *< 0.001	1.45 (1.32–1.59), *P *< 0.001
PKD	0.87 (0.74–1.02), NS	0.99 (0.68–1.45), NS	0.99 (0.83–1.19), NS
SLE	1.89 (1.57–2.29), *P *< 0.001	1.14 (0.84–1.55), NS	1.67 (1.32–2.13), *P *< 0.001
Others	2.09 (1.95–2.25), *P *< 0.001	1.38 (1.25–1.53), *P *< 0.001	1.52 (1.36–1.69), *P *< 0.001

HR, adjusted hazard ratio; CI, confidence interval; GN, glomerulonephritis; PKD, polycystic kidney disease; SLE, systemic lupus erythematosus; and NS, not significant.

**Figure 2:**
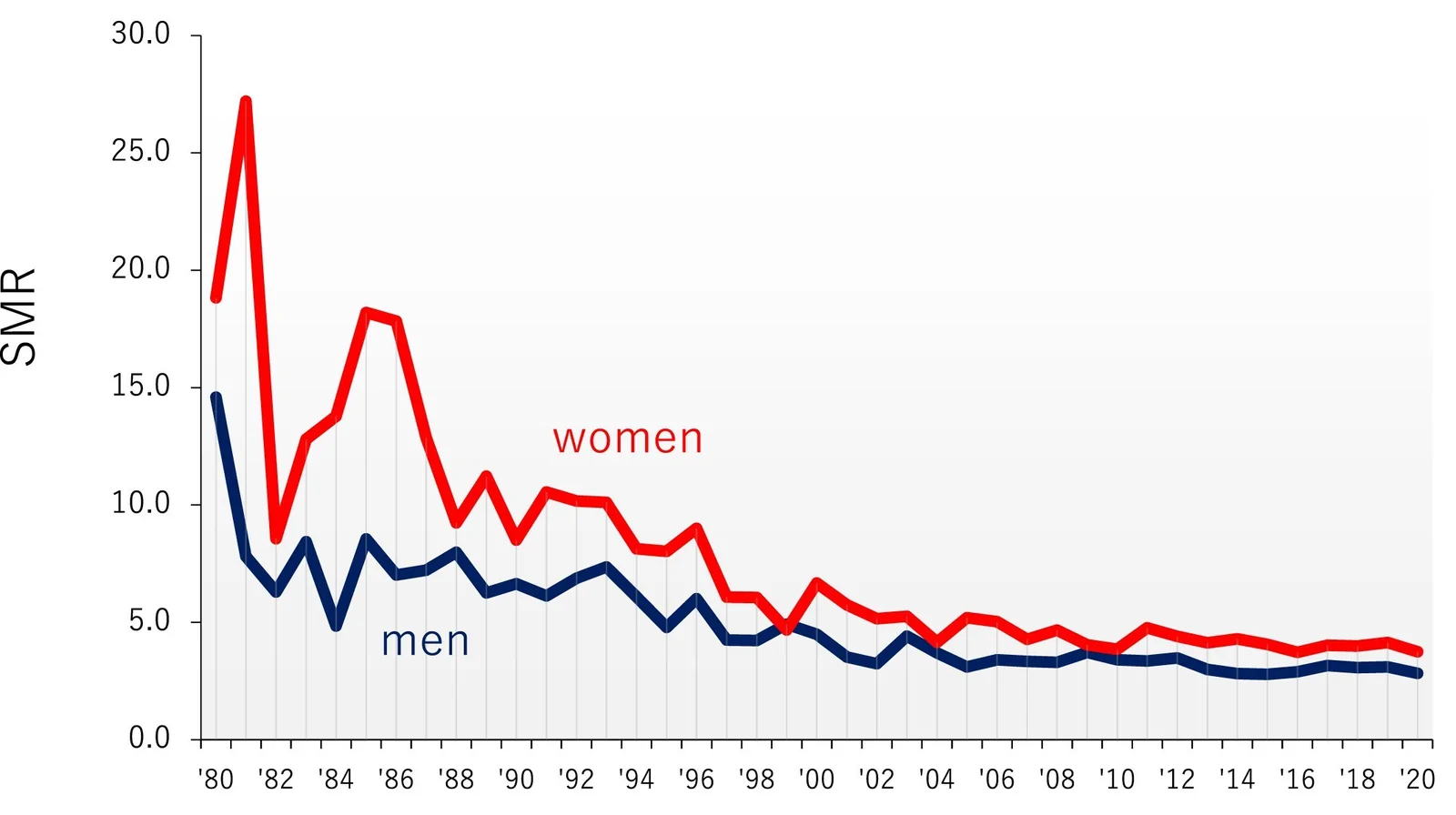
SMR of patients on dialysis. The reference is the data of the general population, for women and men in Okinawa.

SMRs relative to the general Okinawan population declined steeply until the early 2000s, plateaued thereafter, and remained elevated in 2020 at 2.83 for men and 3.75 for women (Fig. 2).

### Differences in survival trends based on dialysis vintage

**Figure 3:**
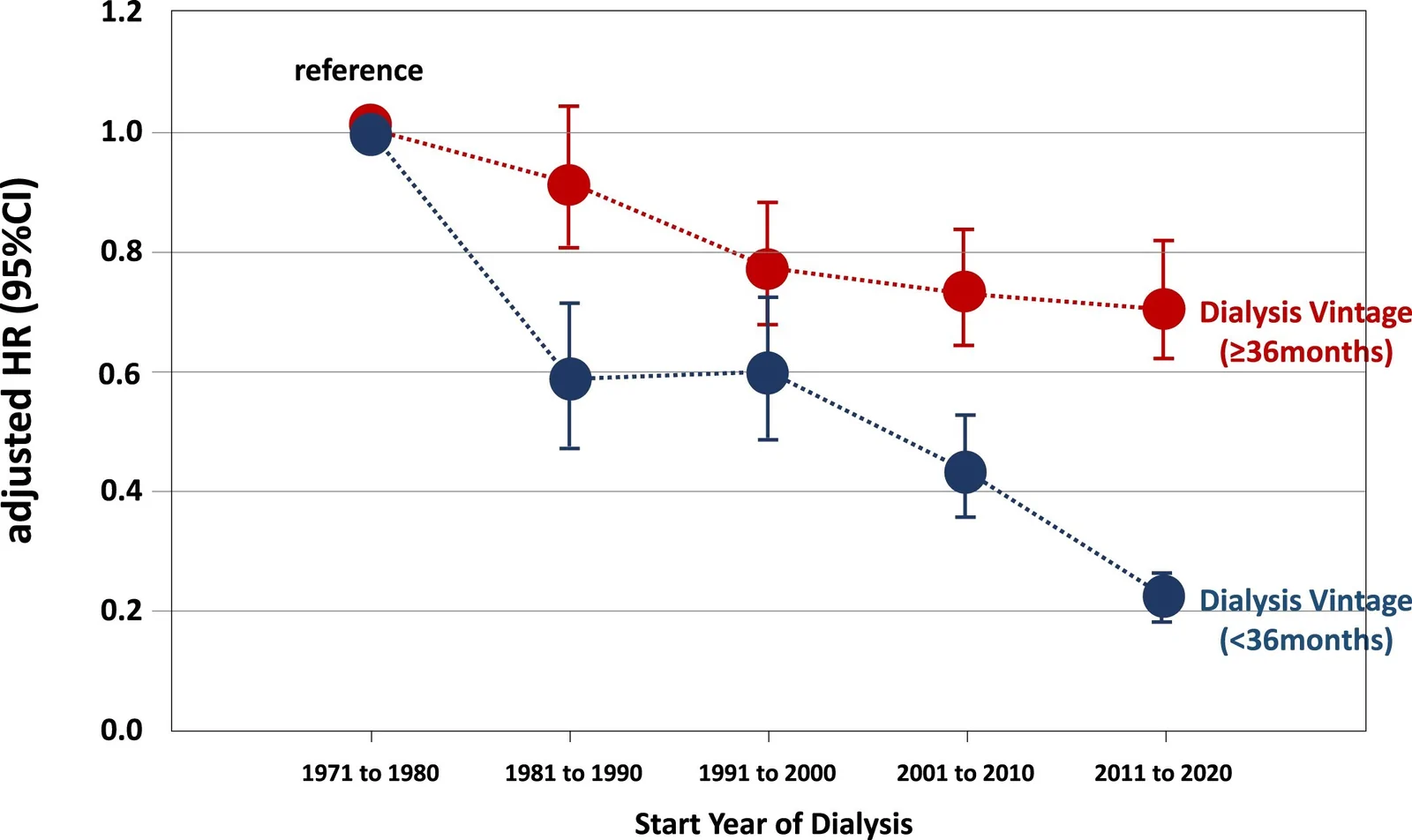
Risk of death by the year in which dialysis was initiated. HR and 95% CIs were adjusted for age, sex, diabetes mellitus, and calendar period of dialysis initiation.

The magnitude of the improvement in mortality differed substantially based on the dialysis vintage. Among patients with a short dialysis vintage (<36 months), survival increased markedly over time, with aHRs declining to 0.21 by 2011–2020, relative to 1971–1980 (Fig. 3).

In contrast, among patients with a long dialysis vintage (≥36 months), improvement in survival was more modest, with aHRs declining only to 0.67 in 2011–2020 over the same time span.

Thus, most survival gains over the past five decades occurred among patients in the early phase of dialysis, whereas improvements among long-vintage patients were comparatively attenuated. After the early 2000s, the separation between the two vintage-stratified mortality curves became more pronounced.

### Trends in standardized mortality ratios

**Figure 4:**
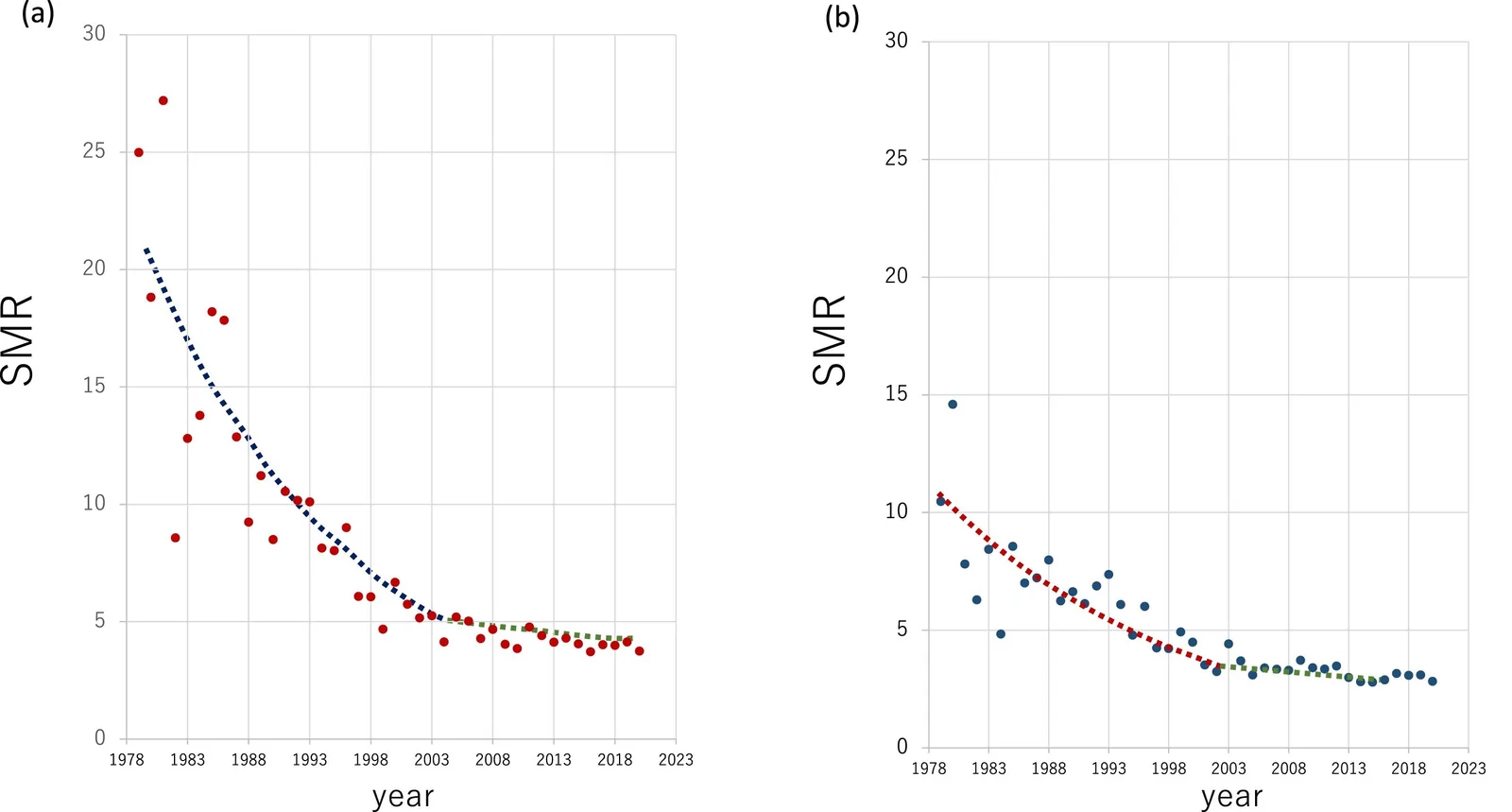
Joinpoint regression analysis of the standard mortality ratio in women (a) and men (b).

Joinpoint analyses demonstrated that SMRs declined significantly from the late 1970s until 2004, after which the rate of decline slowed and no further statistically significant improvement was observed (Fig. 4). This plateau coincided with the period during which improvement in survival among long-vintage patients slowed, whereas gains among short-vintage patients continued.

### Sex-specific patterns

Declines in SMRs were observed in men and women, although the absolute SMRs remained higher in women than in men throughout the follow-up period (Fig. 2).

## DISCUSSION

In this population-based closed cohort study spanning five decades, we observed a marked improvement in survival among patients receiving maintenance dialysis in Okinawa. However, these gains were not evenly distributed across the patient groups. Survival improved far more substantially among patients with a short dialysis vintage (<36 months) than among those with a long dialysis vintage (≥36 months), and the slowing of improvement among long-vintage patients paralleled a plateau in SMRs relative to the general population after the early 2000s. These findings demonstrate that the dialysis vintage is a critical lens through which temporal survival trends should be evaluated.

### Clinical implications of differential survival trends by dialysis vintage

Early after dialysis initiation, the mortality risk is affected by the acute illness burden, vascular access, cardiovascular instability, and access to high-quality dialysis treatment. Improvements in dialysis delivery, infection control, anaemia and bone mineral disorder management, and early care transitions likely contributed to the large survival gains observed among short-vintage patients [12, 13]. Conversely, survival among long-vintage patients improved to a lesser extent, suggesting that the challenges associated with prolonged dialysis exposure, including cardiovascular remodelling, chronic inflammation, protein-energy wasting, frailty, and recurrent infection, remain only partially addressed [14, 15].

Therefore, our results show that overall survival improvement in dialysis populations does not imply equal benefits across the disease course. By separating survival trends based on dialysis vintage, we revealed a shifting composition of risk. As more patients survive the early years of dialysis, a growing proportion are living with the cumulative biological and psychosocial consequences of long-term treatment, a group in whom mortality improvement has slowed [16].

### Factors associated with the improvement in survival

Improved survival among dialysis patients may be partly explained by advances in the management of dialysis-related complications. According to JSDT data, 91.2% of dialysis facilities achieved endotoxin levels below the detection limit of <0.05 EU/ml, and higher endotoxin levels have been associated with increased all-cause mortality [17]. Pharmacological management of CKD–mineral and bone disorder has also evolved over time, with the introduction of vitamin D receptor activators in 1981, calcium-based phosphate binders in 1994, and calcimimetics in 2015. Haemodiafiltration (HDF) has been available in selected units since 2001 [18]. Following reimbursement approval in 2012, its use expanded and was associated with improved survival [19]. In Japan, predilution HDF is commonly used because blood flow rates are generally <250 ml/min. More recently, high-dose HDF has been shown to reduce mortality risk compared with conventional high-flux haemodialysis [20].

The introduction of new therapeutic agents may also have contributed to reducing complications associated with CKD and end-stage kidney disease. These include angiotensin-converting enzyme inhibitors (introduced in 1983), angiotensin II receptor blockers (1998), erythropoiesis-stimulating agents (1990), sodium-glucose cotransporter 2 inhibitors (2014), and hypoxia-inducible factor prolyl hydroxylase inhibitors (2019). In addition, since the publication of the Kidney Disease: Improving Global Outcomes (KDIGO) definition of CKD [21], awareness of CKD has increased worldwide. Alongside advances in dialysis technology and pharmacotherapy, multidisciplinary coordination across subspecialties has become increasingly important in contemporary dialysis care.

### Clinical and health system implications

Our findings have several implications. First, they suggest that historical advances in dialysis care have predominantly benefited patients early in their treatment course. Further improvements in outcomes may increasingly depend on strategies that address late-phase vulnerabilities, including malnutrition–inflammation cachexia, sarcopenia, infection prevention, psychosocial stressors, and treatment fatigue [9, 13, 22, 23]. Accordingly, interventions such as exercise rehabilitation, nutritional optimization, personalized dialysis prescriptions, sleep hygiene, and frailty-focused care may warrant greater attention [24–27].

Second, the plateau in SMRs relative to the general population indicates that, despite substantial progress in dialysis care, population-level mortality among dialysis patients remains markedly elevated. Identifying which patients experience diminishing survival benefits is essential for efficient resource allocation, policymaking, and risk-stratified care planning [28].

Third, vintage-stratified outcomes may serve as quality indicators reflecting how well healthcare systems support patients across the full trajectory of dialysis exposure. Regions that achieve survival gains among long-vintage patients may be more successful in reducing the cumulative complications of chronic dialysis, whereas persistent survival gaps in this group may indicate unmet supportive, nutritional, rehabilitative, or social care needs [29].

In addition to biomedical risk factors, our findings should be interpreted in the context of the ways in which dialysis care has traditionally constrained patients’ everyday lives, particularly their dietary enjoyment [30, 31]. For decades, dietary prescriptions for haemodialysis have emphasized strict restriction of sodium and other nutrients, often requiring patients to avoid culturally familiar and enjoyable foods and to limit social eating. These restrictions may reduce quality of life, contribute to treatment fatigue, and undermine long-term adherence. Although current KDIGO and Kidney Disease Outcomes Quality Initiative guidelines recommend sodium restriction for blood pressure and volume control, they also acknowledge the limited availability of high-quality trial evidence to define the optimal degree of sodium restriction in dialysis populations [32–35].

Recent work in nephrology nutrition has highlighted a gradual shift towards less restrictive and more individualized diet–dialysis policies. Observational studies and qualitative research indicate that rigid dietary rules are difficult for many patients to follow, particularly sodium and fluid restrictions, and that health professionals themselves increasingly question the empirical strength and proportionality of some traditional recommendations [36, 37]. At the same time, emerging evidence suggests that dietary liberalization in selected contexts, such as intradialytic oral intake or extended-hour haemodialysis combined with a more liberal diet, may improve protein–energy status and health-related quality of life, and may possibly improve survival, without clear evidence of harm when volume status and blood pressure are carefully managed [38].

Re-examining restrictive dietary protocols with greater emphasis on enjoyment of eating, individualized sodium targets, and integration of dietary recommendations with dialysis prescriptions may represent an important avenue for improving outcomes among patients living for many years on dialysis.

### Contribution of the Okinawa closed-cohort setting

Several strengths of the Okinawa dialysis network made these observations possible. The geographically isolated island setting enabled near-complete follow-up, minimizing migration-related loss [1, 2]. Long-standing collaboration among dialysis units allowed the reconstruction of patient trajectories over decades, enabling the evaluation of true population-level trends rather than sample-based inferences. This closed-cohort perspective provides a benchmark against which other regions may assess their progress.

### Comparison with prior literature

Previous national and international studies have reported declining mortality among patients on dialysis; however, most analyses have summarized trends without differentiating by dialysis vintage [38]. Our findings expand upon prior work by demonstrating that the trajectory of improvement differs fundamentally between early- and late-phase dialysis, and that overall population-level measures, such as SMR, may obscure this heterogeneity.

### Strengths and limitations

The major strengths of this study include its long observation period, population-based coverage, high completeness of outcome ascertainment, and ability to evaluate temporal changes across clinically meaningful subgroups. Several limitations should also be acknowledged. First, because of the observational design, the associations observed in this study should not be interpreted as causal. Second, diagnostic assignment and coding practices may have changed over the 50-year study period. Third, the granularity of comorbidity and laboratory data was limited, particularly in the earlier years of the registry. Fourth, we were unable to fully account for changes in dialysis practice, pharmacological treatment, and other unmeasured confounders over time.

Another important consideration is the low rate of kidney transplantation in Okinawa. In the present cohort, kidney transplantation was performed in only ∼ 4% of patients throughout the study period. The first kidney transplantation in Okinawa was performed in 1985. In Japan, the Organ Transplant Act was enforced on 16 October 1997, legalizing organ transplantation from brain-dead donors, and the revised Act came into full effect on 17 July 2010, allowing organ donation from brain-dead individuals with family consent when the patient’s own intention was unknown. Despite these legal changes, kidney transplantation has remained relatively limited in Okinawa. This may be related not only to the national organ donation system but also to demographic changes in the dialysis population, including the increasing acceptance of older patients and those with diabetes.

Therefore, direct comparisons of SMRs with those of other regions should be interpreted with caution. In regions with higher transplantation rates, healthier and transplant-eligible patients may be preferentially removed from the dialysis population, resulting in different underlying risk profiles. Such differences in transplantation practices may affect the comparability of dialysis survival and SMRs across regions.

### Clinical and research implications

Our findings highlighted the need to reframe survival improvement in patients undergoing dialysis as a stage-specific challenge. Strategies that have successfully reduced mortality during the early phase of dialysis may now be approaching diminishing returns, whereas patients with prolonged dialysis exposure represent a priority population for future innovation. Therefore, incorporating dialysis vintage-stratified analyses into routine outcome surveillance, inter-regional benchmarking, and clinical trials is essential to avoid masking clinically meaningful disparities. Importantly, for patients living for many years on haemodialysis, further gains in survival may be inseparable from improvements in quality of life. As articulated by Eady, a patient on long-term dialysis, ‘survival is not enough’, emphasizing that longevity without acceptable well-being may fail to represent meaningful success in dialysis care [39]. Recognizing quality of life as a central therapeutic target, rather than a secondary outcome, may be critical for improving adherence, sustaining engagement with treatment, and ultimately enhancing long-term outcomes among patients with an extended dialysis vintage.

## CONCLUSION

Over five decades in a closed-cohort population, mortality among dialysis patients declined substantially; however, survival gains were concentrated among those with short dialysis vintage, whereas improvements among long-vintage patients slowed and paralleled a plateau in SMRs relative to the general population. Recognizing dialysis vintage as a key determinant of temporal survival trends may help guide targeted clinical strategies and quality improvement efforts aimed at improving outcomes for patients undergoing long-term dialysis.

## Data Availability

The data underlying this article will be shared on reasonable request to the corresponding author.
